# The Role of Mitochondria in Brain Aging and the Effects of Melatonin

**DOI:** 10.2174/157015910792246245

**Published:** 2010-09

**Authors:** Germaine Escames, Ana López, José Antonio García, Laura García, Darío Acuña-Castroviejo, José Joaquín García, Luis Carlos López

**Affiliations:** 1 Centro de Investigación Biomédica, Instituto de Biotecnología, Universidad de Granada, Granada, Spain; 2 Departamento de Fisiología, Facultad de Medicina, Universidad de Granada, Granada, Spain; 3 Servicio de Análisis Clínicos, Hospital Universitario San Cecilio, Granada, Spain; 4 Departamento de Farmacología y Fisiología, Facultad de Medicina, Zaragoza, Spain

**Keywords:** Melatonin, mitochondria, oxidative stress, brain, aging, neurodegenerative diseases, neural stem cells.

## Abstract

Melatonin is an endogenous indoleamine present in different tissues, cellular compartments and organelles including mitochondria. When melatonin is administered orally, it is readily available to the brain where it counteracts different processes that occur during aging and age-related neurodegenerative disorders. These aging processes include oxidative stress and oxidative damage, chronic and acute inflammation, mitochondrial dysfunction and loss of neural regeneration. This review summarizes age related changes in the brain and the importance of oxidative/nitrosative stress and mitochondrial dysfunction in brain aging. The data and mechanisms of action of melatonin in relation to aging of the brain are reviewed as well.

## INTRODUCTION: MELATONIN SYNTHESIS AND ITS ACTION'S MECHANISMS

Melatonin is an ancient and highly conserved indoleamine derived from tryptophan. It is present in bacteria, eukaryotes, plants and all phyla of muticellular animals. Because of its ancient origin, it is thought that melatonin posseses numerous functions acquired throughout evolution [59].

The biosynthesis of melatonin requires four enzymatic steps. First, tryptophan hydroxylase catalyzes the conversion of tryptophan to 5-hydroxytryptophan, which is then decarboxylated by aromatic amino acid decarboxylase to produce serotonin. Therefore, the enzyme arylalkylamine *N*-acetyl-transferase (AANAT) converts serotonin to *N*-acetylserotonin, which is then *O*-methylated by the action of the hydroxyindole-*O*-methyltransferase (HIOMT) to produce melatonin [47]. In mammals, melatonin is synthesized by the pineal gland in a circadian manner and it is released into blood and into the cerebrospinal fluid (CSF). In the blood melatonin can concentrate up to 0.5 nM [47, 132].

Blood melatonin concentrations exhibit higher values (10–15-fold increase) during the night than during the daytime. This circadian rhythm is present in all living organisms, from unicellular to multicellular organisms including humans. In vertebrates, the rhythm is generated by a biological clock situated in the suprachiasmatic nucleus (SCN) of the hypothalamus, and synchronized to 24 h primarily by the light–dark cycle acting *via* the SCN [79]. In humans, these rhythms are developed during the first months of life and reach the greatest magnitude between the 4 and 7th year of age. At puberty, there is a drop in melatonin concentrations, and thereafter plasma concentrations diminish gradually. After that, melatonin production in the pineal gland declines progressively with age [79]. As a consequence, in many elderly individuals the day–night differences in melatonin secretion are almost absent. Therefore, in old animals and elderly humans the levels of melatonin available to the organism are a small proportion of that of young individuals [133]. 

In addition to the melatonin produced by the pineal gland, melatonin is also generated in many tissues and organs of the body, and this extrapineal production of melatonin is much more greater than that produced by the pineal [152]. The expression of genes for the key enzymes for melatonin synthesis, AANAT and HIOMT has been found in many organs [146]. Interestingly, melatonin is concentrated by subcellular compartments including nucleus and mitochondria, the latter showing 100-200 times more melatonin than cytosol [5, 95]. 

The multiple sources of melatonin are associated to different indoleamine levels in different tissues, cell types and subcellular compartments and presumably related to the specific actions of the indoleamine in these tissues. Some of these actions are especially remarkable for the potential benefits in the brain, especially during the physiological aging and in pathophysiological age-related disorders: 1) In contrast to numerous synthetic and natural drugs, melatonin is readily administrated orally and it is readily available to the brain [88], 2) numerous *in vitro *and *in vivo* studies have demonstrated the capacity of melatonin to counteract oxidative stress and oxidative damage [7, 134, 135, 136], 3) melatonin reduces chronic and acute inflammation in the brain [6], and 4) melatonin plays an important role in the mitochondrial homeostasis, especially in response to mitochondrial damage [7].

## MITOCHONDRIA, MELATONIN AND AGING BRAIN

The mammalian brain is a tissue with high energy demand and it possess highly active mitochondria metabolism with high oxygen utilization (20% of the total oxygen inspired). Consequently, reactive oxygen species (ROS) generation in the brain is intense. In addition, the brain is very susceptible to free radical damage because of its high concentrations of polyunsaturated fatty acids [48] and transition metals such as iron, which is involved in the generation of the hydroxyl radical [65] , moreover, the brain contains low concentrations of cytosolic antioxidants [40, 134]. Remarkably, it has been shown that the cognitive functions, motor ability, exploratory capacity and neuromuscular coordination in mice are decreased upon aging in parallel with an increase of protein oxidation and a reduction in mitochondrial complex activities in the brain of these animals [49, 114].

Several lines of evidences have shown the relation between aging, increased oxidative damage and mitochondrial dysfunction. Oxidative damage to mitochondria influences different structural and functional components of mitochondrial DNA (mtDNA), proteins and membrane lipids. Three factors make mtDNA particularly vulnerable to reactive species: mtDNA is located close to the mitochondrial inner membrane, near the generation of ROS; mtDNA is not extensively condensed and protected by histones; mtDNA repair is limited [26], the expression of the entire mtDNA is essential for the maintenance of mitochondrial bioenergetic function, while only about 7% of the nuclear genome is expresses during cell differentiation [109].

Age-related mtDNA deletions have been detected in humans in a wide variety of aged tissues including the brain, and the correlation of the rise in mtDNA deletions and mitochondrial respiratory chain malfunction during aging has been amply demonstrated [14, 29, 86, 162]. The global deletion levels rarely exceed 1% and, therefore, the contribution of mtDNA deletions to the aging process appear to be unlikely. However, the deletions levels in particular areas and cell types are still unknown. On the other hand, the mechanisms of mtDNA deletions during aging are still controversial but oxidative damage to DNA associated with single or double-stranded breaks has been proposed. This idea has been supported for several studies: the relative amount of mtDNA deletions correlates with the levels of 8-hydroxy-2'-deoxyguanosine (8-OHdG) [62], and treatment of human skin fibroblasts with sub-lethal dose of oxidative substances and environmental insult inducers of ROS results in the formation and accumulation of the 4977 bp deletion in mtDNA [16, 41].

mtDNA point mutations and duplications in tRNA, protein-coding genes and D-Loop have been also found to accumulate in some post-mitotic tissues during human aging [36, 108, 160]. However, it seems that the proportion of mutant mtDNAs is too low to cause a significant impact on mitochondrial function in aging tissues. On the other hand, the distribution of the mutant mtDNA in cells and tissues is still unknown and clarification of this matter could resolve important questions regarding the importance of mtDNA mutations in aging [99]. Additionally, mitochondria polymerase γ (POLG) deficient mice accumulate high levels of mtDNA mutations and deletions resulting in a premature aging phenotype with COX deficient cells in the brain and heart but without an increase of ROS generation and oxidative damage [87, 156, 159]. Thus, these results support the idea of a direct involvement of mtDNA mutations in aging but cast doubt on the vicious cycle theories of aging and oxidative stress [93].

Some explanations have been proposed, however, to account the lack of oxidative stress in POLG deficient mice: aging is the result of alterations in many pathways and, therefore, the aging phenotype in the mutator mice should not be compared to normal aging; alterations in POLG may be downstream from mechanisms that generate ROS; and extensive and equally distributed mtDNA mutations (in contrast to the mosaic distribution of mtDNA mutations observed in normal aging) could prevent the generation of ROS [93]. In contrast to POLG mutant mice, skin fibroblasts harboring mtDNA point mutations associated with aging show an alteration in the expression profile of antioxidant enzymes [161].

In addition to the research related to mtDNA alterations, mitochondrial protein oxidation has been also reported in a variety of organisms during aging [99]. The proteins containing Fe-S clusters seem to be the most susceptible to oxidation [99]. Several reports have revealed that oxidation of aconitase, adenine nucleotide translocase and mitochondrial respiratory chain complexes may occur during aging and consequently the activities of these enzymes diminish with increasing age [9, 168, 169]. Oxidative injury is not limited to mtDNA or proteins but also to mitochondrial membranes. This may lead to a progressive lipid peroxidation (LPO) and cross linking damage, with simultaneous changes in the respiration rate, ATP synthesis, membrane fluidity and permeability, Ca^2+^ homeostasis and apoptosis [3].

Alterations in the expression and activities of the antioxidant enzymes in response to the oxidative environment in the aging cells has been found in human blood [10, 33, 57] and muscle [102, 121], and in a variety of tissues from rats and mice, including skeletal muscles, brain and heart, which are tissues with high energy demand. Additional information about the physiological changes in mammalian aging has been uncovered by studies performed in the senescence-accelerated mouse (SAM) [150]. SAM includes two strains, one prone to accelerated senescence (SAMP) and one resistant to accelerated senescence (SAMR). SAMP8, a sub-strain of SAMP, shows relatively strain specific age-associated phenotypic pathologies such as a shortened life span and early manifestation of senescence (including loss of activity, alopecia, lack of hair glossiness, skin coarseness, periphthalmic lesions, increased lordokyphosis and systemic senile amyloidosis), similar to several geriatric disorders observed in humans [148, 150]. SAMP8 mice show a general hyperoxidative status manifested by increased mitochondrial electron leakage and ROS production, elevated LPO and protein carbonyl content, changes in the antioxidant enzymes activities and increase of GSSG:GSH ratio [23, 68, 92, 105, 117, 149, 171]. The results are a loss in the mitochondrial respiratory chain activity, ATP synthesis and energy status of the organism, suggesting that the mechanism of senescence acceleration in SAMP8 mice is related to free radical damage [138,139]. SAMP8 mice also show an age-dependent increase in IFN-γ and TNF-α,  a reduction in IL-2 levels and an rise in nitric oxide (NO•) levels [140], suggesting the existence of an inflammatory process during aging. The increase of NO• levels is particularly relevant since this molecule reacts with (O_2_^._^) in mitochondria yielding peroxynitrite anion [126], which irreversible impairs the mitochondrial respiratory chain and decreases the efficiency of oxidative phosphorylation, leading to energy depletion and cell death [20, 25].

Numerous studies have shown that melatonin directly scavenges free radicals, stimulates other antioxidant systems of the cells and inhibits the expression of iNOS with the subsequent reduction in the levels of both ROS and RNS [3, 6, 8]. In recent studies, Jou and colleagues [73, 74] demonstrated that cybrids with 80% common deletion in mtDNA showed an increase in oxidative stress and an rise in susceptibility to a secondary oxidative stress induced by H_2_O_2_ exposure. Interestingly, melatonin reduced ROS generation in both conditions preventing ROS-mediated depolarization of mitochondrial membrane potential and subsequent opening of the mitochondrial permeability transition pore (MPTP) and cytochrome c release [74]. The protection provided by melatonin was superior to other antioxidants such us vitamin E and mitochondria targeted coenzyme Q (MitoQ) [74]. 

The effects of melatonin in counteracting oxidative damage and mitochondrial dysfunction have been also tested in the brain of mice with accelerated aging, SAMP8 [24]. Chronic melatonin administration in the drinking water for 9 months (10 mg/kg b.w.) completely prevented the mitochondrial impairment maintaining or even increasing ATP production. Likewise, melatonin prevented the rise in mitochondrial LPO and increased GPx and GRd activities normalizing the GSSG/GSH ratio [24]. Melatonin also counteracted the oxidative damage to the mitochondrial membranes of the brain during aging since it prevented rigidity in the mitochondrial membrane and preserved the content and structural integrity of cardiolipin molecules [54, 125]. Immune function in the brain is other important factor in aging. Nitrite levels accurately reflect the nitrosative stress status that is caused by inflammation. Importantly, age-dependent nitrosative status in brain mitochondria was prevented by melatonin administration [24]. The effect of melatonin in the reduction of nitrosative stress has been amply studied in animal models of sepsis. The administration of pharmacological doses of melatonin in rodents with sepsis induced by lipopolysacharide injection or cecal ligation and puncture (CLP) produced a decrease in the expression and activity of iNOS, and consequently nitrite levels, nitrosative/oxidative stress and mitochondrial function were normalized [42, 43, 45, 96, 97]. Interestingly, the increase of iNOS expression was more pronounced in aged rats (18 m.o.) than young rats (3 m.o.), but melatonin was able to reduce the expression to the same levels in both groups [42, 44].

## MITOCHONDRIA, MELATONIN AND NEURODEGENERATIVE DISORDERS

Aged-related disorders include neurodegenerative diseases of different etiologies that may share mitochondrial dysfunction, oxidative/nitrosative stress and apoptosis in particular brain areas as a final common pathway. Consequently, neuronal loss may be associated to mitochondrial dysfunction in those disorders. Alzheimer disease (AD) is a predominantly sporadic late-onset disorder characterized by progressive dementia with a relatively long course. Progressive neuronal loss, particularly in the cortex and the hippocampus, is typically observed in the brain of Alzheimer patients. The two main histopathological features of AD are the accumulation of extracellular neuritic plaques, mainly represented by β-amyloid (Aβ), and of neurofibrillary tangles, mainly represented by the hyper-phosphorylated forms of the microtubule-associated protein tau [56]. Additionally, some evidence indicates that mitochondria are involved in the pathology of AD, including a reduction in brain energy metabolism shown by positron emission tomography [11], defects of mitochondrial metabolic enzymes [104, 144], mitochondrial respiratory chain complex deficiencies [19, 85] and an increase of mtDNA deletion level in the substantia nigra neurons [15]. On the contrary, a recent review found little evidence in support a role of mtDNA mutations in the development of AD [69]. 

It has been shown that β-amyloid peptide generates ROS in a metal-catalyzed reaction, which induces neuronal cell death in a ROS-mediated process resulting in damage to neuronal membrane lipids, proteins and nucleic acids. This suggests that the use of antioxidants such as vitamin E, melatonin or estrogens may be beneficial in AD [90, 110]. In AD patients, melatonin levels are reduced in blood and CSF and that reduction seems to go in parallel to the progression of AD neuropathology [165, 174]. Moreover, CSF melatonin levels are already decreased in pre-clinical AD individuals [165].

The administration of melatonin has been tested in order to reduce the neurodegenerative manifestations in AD [123]. When neuroblastoma cells were incubated with Aβ, more than 80% of the neurons died due to apoptosis, but the presence of melatonin reduced cellular death and DNA damage in a dose-related manner [124]. In human platelets, melatonin also protected against Aβ-induced damage [21, 122]. Recently, melatonin treatment has been tested in the APP + PS1 double transgenic (Tg) mouse, which is considered a mouse model with characteristics of the neuropathology of AD [119]. Melatonin administred in the drinking water (100 mg/L water) for four months protected AD mice from cognitive impairment in a variety of tasks of working memory, spatial reference learning/memory, and basic mnemonic function. Immunoreactive Aβ deposition was significantly reduced in hippocampus (43%) and entorhinal cortex (37%) of melatonin-treated AD mice. The levels of tumor necrosis factor (TNF)-alpha were reduced in the hippocampus of AD mice treated with melatonin, as well as the cortical mRNA expression of SOD-1, GPx and catalase. Taken together, the results suggest that melatonin's cognitive benefits could involve its anti-Aβ aggregation, anti-inflammatory, and/or antioxidant properties [119]. In AD patients, melatonin stabilizes cognitive function over a 2–3 year period [21, 22]. An additional retrospective study reported that individuals with mild cognitive impairment given melatonin for sleep enhancement also showed significantly better cognitive performance in two widely utilized cognitive assessment tests [52].

Parkinson disease (PD) is a mainly sporadic late-onset disorder characterized by bradykinesia, rigidity and tremor. PD is accompanied by the loss of about 60% of dopaminergic neurons of the substantia nigra pars compacta. Mitochondrial involvement in PD is suggested by deficiencies of complex I (C-I) in substantia nigra [142], with a parallel reduction in GSH levels, suggesting the existence of oxidative stress. In platelets of PD patients, C-I is also decreased and in some cases is accompanied by complex II (C-II), complex III (C-III) and complex IV (C-IV) deficiencies.

Mitochondrial involvement in the pathology of PD has been genetically supported by the finding of *POLG* mutations in early-onset Parkinsonism in different families [32, 98]. In some cases, the *POLG* mutations were accompanied by mtDNA deletions, ragged-red and cytochrome c oxidase-negative fibers and low activities of mitochondrial complexes containing mitochondrial DNA-encoded subunits [32, 98]. Additionally, analysis of single substantia nigra neurons from individuals with PD and age-mathed controls showed an age-related accumulation of high levels of mtDNA deletions [14]. On the contrary, a recent review of the evidence for primary mtDNA mutations in PD led to the conclusion that there is no convincing proof for a primary role of mtDNA mutations in this neurodegenerative disorder [69]. 

A series of nuclear genes (*PARK2*, *PARK7*, *PINK1*, *SNCA*, *LRRK2* and *HTRA*2) are recognized to be associated with the familial form of PD and the proteins encoded by these genes interact directly or indirectly with mitochondria and seem to be involved in apoptosis [35]. Moreover, some environmental toxins seem to interact with the products of these genes, which provoke oxidative damage, mitochondrial dysfunction and cell death [2]. These environmental toxins influence PD, as shown by the C-I inhibitory effects of 1-methyl-4-phenyl-1,2,3,6-tetrahydropyridine (MPTP), rotenone and paraquat. The C-I inhibition is prevented by free radical scavengers indicating oxidative damage to C-I. Moreover, MPTP also stimulates NMDA-dependent nNOS activity thereby increasing NO• production [155], and decreasing the content of mtDNA [111]. In mouse models of PD induced by MPTP melatonin administration normalized complex I activity and oxidative status in mitochondria from substantia nigra and striatum.

Looking for the targets of melatonin action, it was recently shown that this indole reduced the activity of the mitochondrial iNOS (i-mtNOS), thus decreasing mitochondrial NO• levels and preventing the respiratory inhibition produced by NO• at the level of complex IV [153]. Melatonin also protects against excitotoxicity by reducing the autoxidation of dopamine (DA) which occurs in PD [80]. These effects were demonstrated in MPTP-induced PD in mice [4, 120] and in PC12 cells incubated with 6-hydroxydopamine [106]. Melatonin also abrogated cell death induced by cysteamine pretreatment of the PC12 cells; cystamine treatment involves mitochondrial iron sequestration [50]. 

The age-associated accumulation of redox-active iron in subcortical astrocytes may facilitate the bioactivation of DA to neurotoxic free radical intermediates and thereby predispose the nervous system to PD and other neurodegenerative diseases. In rats injected with kainic acid to produce excitotoxicity-induced apoptotic cell death, melatonin significantly attenuated apoptosis, an effect linked to the reduction in oxidative damage and an increased GSH content [27]. In a spontaneous, age-induced model of apoptosis using cerebellar granule cells, it was shown that melatonin and Ca^2+^-channel blockers such as amlodipine, inhibited spontaneous apoptosis [103]. This antagonism between melatonin and Ca^2+^-channels was also demonstrated in electrophysiological and binding experiments [46]. Striatal neurons growing in low density culture in serum-free medium and in the absence of glia die within 3 days by apoptosis. The presence of melatonin rescues striatal neurons from impending cell death, which has important consequences in neurodegenerative diseases involving nigrostriatal pathway such as in PD [71]. 

Huntington’s disease (HD) is a neurodegenerative disorder characterized by ataxia, chorea and dementia. It is known to be caused by an alteration in a gene for nDNA encoding huntingtin, a widely expressed protein of unknown function but associated with inappropriate apoptosis. The pathology of HD involves mainly the GABA-containing neurons of the caudate nucleus and putamen [142]. Excitotoxicity has been suggested to play an important role in this disease. This includes activation of NMDA-dependent neuronal nitric oxide synthase (nNOS) and NO• production. NO• and particularly peroxynitrite mediate oxidative damage. Increases in mtDNA copy number and multiple mtDNA depletions have been found in HD patients, which are especially common in the frontal and temporal lobes of the cerebral cortex, although its significance is unclear [67, 130]. These mtDNA alterations could be a consequence of the increase of the oxidative damage to DNA reflected by an elevation of the levels of 8-hydroxydeoxyguanosine [130]. There are also deficiencies in the activities of C-II, C-III and C-IV in caudate and in a lesser extend in putamen in HD.

Others mitochondrial abnormalities have been found in cells and animals models of HD, which include calcium dyshomeostasis and anomalous mitochondrial dynamics [130]. Melatonin treatment has been used in a rat model of Huntington disease induced by 3-nitropropionic acid, a mycotoxin that inhibits the mitochondrial succinate dehydrogenase or complex II [157]. The inhibition of complex II by 3-nitropropionic acid was accompanied by rises in LPO and protein carbonyl content, and a decrease in SOD activity in the brain cortex and striatum. Melatonin administered intraperitoneally (1 mg/kg b.w./day) for 8 days prevented the deleterious effects induced by the acid [157].

Amyotrophic lateral sclerosis (ALS) is a late-onset sporadic disorder clinically characterized by progressive muscle weakness, atrophy, spasticity widespread paralysis and premature death. The disease is caused by the degeneration and death of upper and lower motor neurons in the cortex, brainstem, and spinal cord [64]. About 5%–10% of patients have a familial form of ALS (FALS), and about 20% of these harbor mutations in the *SOD1* gene that encodes the Cu,Zn-superoxide dismutase 1 (SOD1) [64]. Mouse models overexpressing mutant SOD1 also develop motor neuron degeneration. Most pathogenic mutations do not impair SOD1 activity, and some studies have proved that a portion of mutant SOD1 is localized in mitochondria, both in FALS patients and in the animal models. As a result, it has been hypothesized that mutant SOD1 may damage mitochondria by some misunderstood mechanism [64].

A recent investigation has used melatonin in cellular and mouse models of ALS, as well as 31 ALS patients [163]. First, NSC-34 cells, a widely used motoneuron-neuroblastoma fusion line, were exposed to 2 or 10 mm of glutamate for 3 days. A mortality of 29.2% and 52.1% of cells were detected, respectively; treatment with 50 mM melatonin recovered the survival by 17.2 % (2 mM glutamate) and 8.5 % (10 mM glutamate). Melatonin reduced the elevated ROS production in this cellular model. Second, in the SOD1^G93A^-transgenic mouse (an ALS mouse model), melatonin was administrated in the drinking water (0.5 mg/ml water) at 28 days old, resulting in a delayed disease progression and extended survival; third, daily doses of 300 mg melatonin were administrated in ALS patients as novel suppositories at bedtime. Melatonin treatment decreased the serum protein carbonyls compared with the elevated levels presented in the serum of the matched untreated ALS patients [163].

## NEURAL STEM CELLS IN AGING AND AGED-RELATED DISORDERS: THE IMPORTANCE OF MITOCHONDRIA AND OXIDATIVE STRESS

Stem cells can be classified in three types: pluripotent embryonic stem cells (ESC) that have the potential to differentiate into any cell type in the organism; multipotent cells derived from adult tissue including umbilical cord blood and amniotic fluid, which can differentiate into a limited number of cells types of their own lineage, e.g., mesoderm; and precursor cells, which are adult stem cells committed to differentiation. In the brain, both neural stem cells and neural progenitor cells are responsible for neurogenesis. Neural stem cells produce additional stem cells as well as offspring that go on to differentiate into oligodendrocytes, other glial cells and neurons. Neural progenitor cells have a limited replicative potential that are committed to the neuronal lineage [175]. The vast majority of cells in the adult central nervous system (CNS) are generated during the embryonic and early postnatal period, but some proportion of neurogenesis is also occurs during adulthood. The functional significance of the adult neurogenesis is not fully understood but it has been shown to be involved in several brain function and pathologies [38, 175]. Neurogenesis in the adult brain from mammals is concentrated in the subventricular zone (SVZ) of the lateral ventricule wall and the subgranular zone (SGZ) of the dentate gyrus of the hippocampus [175]. New cells generated from these regions migrate toward their final destinations, where they differentiate into mature cells and are integrated into the CNS [76, 175].

Age-related changes have been observed in both SVZ and SGZ, which are associated with a decline of neurogenesis during aging [38, 175]. These changes may affect the different steps on neurogenesis: 1) proliferation of new cells; 2) survival of newly born cells; 3) migration of these cells toward target areas; and 4) differentiation into mature functional cells.

Neural stem cell proliferation has been widely studied in rodents by use of cell proliferation markers such as bromo-deoxyuridine (BrdU) and tritiated thymidine [76, 175]. Most results have shown that cell proliferation declines during aging in both SVZ and SGZ [76, 175]. However, the difference in the proliferation decline is not clear between middle age and senescence since some studies have reported significant changes in both ages groups [18, 129] but others did not [39, 128].

On the contrary, the short-term survival pattern in the newborn cells seems to be unaffected by aging [38]. However, when some brain areas are damaged, the survivals of newborn cells show clear differences between young and old animals. In one study of stroke simulation, rats subjected to ischemia showed an induction of neural stem cell proliferation in the SGZ. One day after the ischemia induction young adults exhibited a 5.7 fold increase in BrdU labeled cells compared to a 10.6-fold increase in old adults. Remarkably, 65.5% of the labeled cells survived 28 days after ischemia in young adults, whereas in old adult brains only 15.3% remained [167].

In other study, Zaman and Shetty [173] observed that when hippocampi was first damaged by the action of kianic acid, a specific agonist for kainite receptors with neuroexcitotoxic and epileptogenic properties, the survival of rat fetal hippocampal cells injected into the ventricles of rats was 30% in middle-aged and old brains compared with the 72 % survival in young brains. Both studies suggested that changes in the environment of the brain areas during aging may be critical for the survival of new cells [167, 173]. Thus, the increases of glucocorticoids and the decreases of the levels of Insulin-like growth factor 1 (IGF-1) in aged brain have been associated to a decrease in neurogenesis [1, 91, 115].

Interestingly, oxidative stress, which is particularly increased in the rat hippocampus during aging [116], also may play an important role in neural and progenitor stem cell survival. It has been shown that curcumin, an antioxidant and anti-inflammatory agent, modulates the proliferation of embryonic neural progenitor cells with a biphasic effect in cultured cells. In mouse brain, curcumin administration resulted in a significant rise in the number of newly-generated cells in the SGZ of hippocampus [82]. Oxidative stress disrupts the differentiation of oligodendrocyte precursors and neural progenitor cells into mature oligodendrocyte and neurons [51, 143]. On the contrary, the increase of the antioxidant capacity protects neural progenitor cells *in vitro* and potentiates the formation of cellular networks that provide neuroprotection *in vivo *[100, 143].

An increase in newly generated neural cells in adult brain and an increase in the resistance of neurons to dysfunction and apoptosis have been detected in rodents undergoing dietary restriction. Both oxidative stress and dietary restriction are interlinked and related to mitochondrial function. However, few studies have focused in the role of mitochondria in proliferation, survival and differentiation of neural stem cells, and most of them are related with the mitochondrial apoptotic pathway induced by some toxic agent [77, 78,151, 154].

Interestingly, some studies in embryonic stem cells (ESC) have focused on the activity of mitochondrial genome and of these data could be extrapolated to neural stem cells. Transmitochondrial embryonic stem cells harboring pathogenic mtDNA mutations have shown to be compromised in neuronal differentiation when the mitochondrial respiratory chain function is severely affected [84]. Thus, some authors have hypothesized that stem cell competence may be verified using functional mitochondrial characteristics [94]. Differentiation of mouse and human embryonic stem cells (ESC) results in changes in mitochondrial structure, morphology and pattern of cytoplasmic localization. Mitochondria in stem cells tend to localize perinuclearly [94]. Moreover, ESC have relatively few mitochondria with poorly developed cristae [28, 118], and restricted oxidative capacity. As cells are allowed to differentiate, the number of mtDNA copies increase and these differentiated cells contain elevated numbers of mitochondria with distinct cristae, dense matrices and high membrane potentials. These features suggest the initiation of metabolic activity through oxidative phosphorylation (OXPHOS) [145]. Because ESC display low oxygen consumption and thus, poor OXPHOS, an elevation in ATP content per cell may therefore reflect a loss of stemness and the subsequent onset of differentiation [28, 94]. Therefore, preservation of immature mitochondria with a perinuclear arrangement, reduced expression of OXPHOS enzymes and low metabolic activity in ESC has led to the suggestion that these mitochondrial properties might be important for the maintenance of pluripotency and should be considered as another ESC marker. Departures from this profile indicate that cells are differentiating or perhaps becoming senescent.

The increase in mitochondrial mass is accompanied by elevated ATP production and, thus, by a greater generation of ROS. Undoubtedly, the intracellular levels of ROS are higher in differentiated than in undifferentiated ESC, due to the increase in OXPHOS metabolism in the former [141]. An increase in ROS levels might have a role in cell signaling and regulation of proliferation and differentiation. Exposure to low levels of ROS has been reported to enhance ESC differentiation whereas continuous exposure to high levels of ROS results in inhibition of differentiation [141]. Therefore, differentiating cells probably activate effective antioxidant systems, including catalase, GPx and others. In summary, successful differentiation of embryonic cells *in vivo* or ESC *in vitro* involves initiation of mtDNA transcription and replication, an increase in the number of mitochondria, and regulation of the enzymes required for aerobic metabolism in order to fulfill the elevated ATP requirements of fully differentiated cells.

The migration and differentiation of neural and progenitor stem cells may also be affected with aging. It has been reported that the capacity of the newly born cells to migrate from SGZ to the granule cell layer is decreased with aging [63]. Several studies have also shown different grades of reduction in the differentiation of neural stem cells into neurons [38] and the reduction in the dendritic maturation during aging [38, 129].

In addition of the alterations in neurogenesis during physiological aging, some insults can also dramatically affect this process. Neurodegenerative diseases are characterized by loss of neurons and newly-generated neurons should appear in the damaged area to repair this injur. However, recent studies have documented alterations in neurogenesis in neurodegenerative diseases. Cell proliferation is significantly repressed in SVZ of PD patients and animals models of PD, resulting in a decrease in the numbers of neural stem cells and neuroblasts. Importantly, this reduction is more significant in PD patients with cognitive impairments than in those without [66]. AD is characterized by the accumulation of Aβ that suppresses the proliferation of neural stem and progenitor cells and the neuronal differentiation in cell culture [60]. Mouse models of AD with accumulation of Aβ and mice given Aβ intravenously show a defect in neuronal production, survival and differentiation in the SGZ, as well as migration of neuroblasts [37, 61, 164]. Another proof of the involvement of neural stem cell in the pathology of AD is the fact that long term administration of cholinesterase inhibitors, which improve cognitive function in AD patients, promotes the survival of newly-generated neurons and increases neurogenesis in adult mice [75]. The pathophysiology of HD includes atrophy of the caudate nucleus and putamen, which are adjacent to the SVZ. Related to that, Curtis and colleagues [30, 31] observed an enhanced thickness of the SVZ together with an increase of cell proliferation in the brain of HD patients. Subsequently, Batista and colleagues [13] reported that the ability of neural stem cells dissociated from the SVZ of the R6/2 mice, a mouse model of HD, to self-renew increases in parallel with the progression of the disease. Likewise, they observed the presence of migrating neuroblasts and newly-generated neurons in the striatum of these mice. However, the migration of neuroblasts toward the olfactory bulb was significantly suppressed [13]. Contrary to the SVZ, cell proliferation and neurogenesis of the SGZ are decreased in the mouse models of HD [55, 89], while the relation of these findings with the neuropathology of DH has not been clarified.

Independently of the involvement of neurogenesis in the pathology of neurodegenerative diseases, stem cell research focused in the cell replacement therapy is a promising treatment for neurodegenerative disorders. Different strategies are currently being examined for the treatment of neurodegenerative disorders using neural stem cells, including approaches involving transplantation of exogenous cells or promoting proliferation of endogenous cells. In both cases it is believed that the increase in neural stem cells in the brain attenuates anatomic and functional deficits associated with diseases of the CNS *via* cell replacement, release of specific neurotransmitters and production of neurotrophic factors that protect injured neurons and promote neuronal growth.

In mouse models of HD, the administration of basic fibroblast growth factor (bFGF) increased the SVZ cell proliferation and increased migration of neuroblasts to the striatum and the regeneration of the striatal projection neurons [72]. The result was an amelioration of the motor dysfunction and the increase of the life-span of these mice. The rise of hippocampal neurogenesis by the enrichment of the mouse environment also delayed the progression of HD symptoms in the mouse model [158]. Recently, mouse neural stem cells were transplanted intraventricularly into R6/2 HD mouse model combined with dietary trehalose, which reduces cellular aggregate formation. The combined treatment resulted in an improvement of motor function, reduction in aggregate formation and increase of the life-span of the animals [170]. However, the information related to the migration and the survival of the grafted neural stem cells was not provided in this study [83]. Previously, it was shown that fetal cell transplantation ameliorated neuronal dysfunction and improved motor function in both, the HD mouse model and HD patients [12, 113]. 

Stem cell transplantation has been examined in animal models of ALS. Transplantation of neural stem cells isolated from fetal spinal cord or neurons generated from the NT2 human teratrocarcinoma cell line in the spinal cord of ALS mice were effective in the functional improvement and in the delay of the progression of the disease [53, 166]. The transgenic SOD^G93A^ mouse model of ALS was transplanted with human neural stem cells overexpressing vascular endothelial growth factor (VEGF) resulting in a functional improvement and extended survival. The immunohistochemical analysis demonstrated that the transplanted neural stem cells migrated into anterior horn of the spinal cord and differentiated into motoneurons [70]. Thus, a clinical trial of mesenquimal stem cell transplantation in ALS patients has recently finished phase I and is currently underway on phase II [107].

Recently, the effect of neural stem cell transplantation has been evaluated in mouse model of AD (3xTg-AD) which express pathogenic forms of amyloid precursor protein, presenilin, and tau [17]. The results showed that hippocampal neural stem cell transplantation rescues the spatial learning and memory deficits in aged 3xTg-AD mice. However, these improvements were not associated with an alteration of Aß or tau pathology. Interestingly, the mechanism underlying the improved cognition involves an augmentation of hippocampal synaptic density, mediated by brain-derived neurotrophic factor (BDNF) [17]. To further confirm this result, aged 3xTg-AD mice were treated with recombinant BDNF, which mimicked the beneficial effects of neural stem cell transplantation. On the contrary, depletion of neural stem cell-derived BDNF failed to improve cognition or restore hippocampal synaptic density [17].

Transplantation of human fetal ventral mesencephalic cells into the striatum of PD patients has been carried out since early 1990s in patients with advanced disease [83]. However, the evidence of poor survival of the transplanted cells in the brain together with the difficulties to obtain enough fetal tissue for transplantation have led to a redesigned stem cell therapeutical strategy in PD [58, 83]. Thus, dopaminergic neurons have been generated from embryonic stem cells, mesenchymal stem cells and neural stem cells following different experimental protocols [83]. Dopaminergic neurons generated from monkey embryonic stem cells and human neural stem cells have been transplanted into striatum of monkeys with PD induced by MPTP. The results showed a behavioral improvement of the PD monkeys [131, 147]. Transplantation of immortalized neural stem cells into the striatum of a rat model of PD also induced functional improvement [172]. Other strategies include the transplantation of neural stem cells transfected with specific genes such as tyrosine hydroxylase (TH) and GTP cyclohydrolase I (GTPCH1) [83].

The use of stem cells for therapeutical purposes is a promising strategy but several issues must be clarified before of general use in clinical medicine. The concerns include: 1) it must be verified which type of stem cell is most suitable for each purpose; 2) stem cells that escape differentiation and selection processes might expand and produce tumor in the graft site following transplantation; 3) highly purified populations of neural cell types derived from embryonic or neural stem cells may contain other neuronal or glial cells types that could generated unpredictable interactions among grafted cells or host cells; and 4) earliest studies have demonstrated that the long-term survival and phenotypic stability of stem cell-derived neurons or glial cells in the graft following transplantation are unsuccessful [83]. Hence, it is necessary to establish which factors are involved in the poor survival and stability of the transplanted cell. Among them, the highly toxic oxidative and nitrosative environment in the aged brain and its interaction with mitochondrial function should be taken into account.

## NEURAL STEM CELLS AND MELATONIN

Whereas a role of mitochondria in stem cell proliferation and/or differentiation begins to have experimental support, the role of melatonin remains unclear. One can presume that, in view of the specific and significant effects of melatonin on mitochondrial physiology, the indoleamine may also affect mitochondrial physiology in stem cells. It was recently reported that melatonin modulates the proliferative and differentiative ability of the neural stem cells from fetal mouse brain in a concentration and exposure-timing dependent manner [112]. When pharmacological concentrations of melatonin (1-100 µM) were applied during the proliferation period, the proliferation was diminished. Interesting, neural differentiation of these cells increased without affecting astroglial differentiation. Other data point towards a net hippocampal neurogenesis in adult mice by melatonin [127]. Interestingly, it was shown that pinealectomy causes loss of pyramidal neurons in rat CA1/3 hippocampal layers, an effect reversed by melatonin administration [34]. Melatonin also promotes neurogenesis and motor recovery after mild focal ischemia or cranial irradiation in mice [81, 101]. New experimental data suggest that melatonin induces neurogenesis in dentate gyrus of adult pinealectomized rats [137]. The effects of melatonin on neural proliferation and differentiation might be partly a result of melatonin´s activity in mitochondria. Clearly, additional studies are required to uncover the underlying actions of melatonin on neural stem cells.

## References

[1] Aberg MA, Aberg ND, Hedbacker H, Oscarsson J, Eriksson PS (2000). Peripheral infusion of IGF-I selectively induces neurogenesis in the adult rat hippocampus. J. Neurosci.

[2] Abou-Sleiman PM, Muqit MM, Wood NW (2006). Expanding insights of mitochondrial dysfunction in Parkinson's disease. Nat. Rev. Neurosci.

[3] Acuna CD, Escames G, Carazo A, Leon J, Khaldy H, Reiter RJ (2002). Melatonin, mitochondrial homeostasis and mitochondrial-related diseases. Curr. Top Med. Chem.

[4] Acuna-Castroviejo D, Coto-Montes A, Gaia Monti M, Ortiz GG, Reiter RJ (1997). Melatonin is protective against MPTP-induced striatal and hippocampal lesions. Life Sci.

[5] Acuna-Castroviejo D, Escames G, Leon J, Carazo A, Khaldy H (2003). Mitochondrial regulation by melatonin and its metabolites. Adv. Exp. Med. Biol.

[6] Acuna-Castroviejo D, Escames G, Lopez LC, Hitos AB, Leon J (2005). Melatonin and nitric oxide: two required antagonists for mitochondrial homeostasis. Endocrine.

[7] Acuna-Castroviejo D, Escames G, Rodriguez MI, Lopez LC (2007). Melatonin role in the mitochondrial function. Front. Biosci.

[8] Acuna-Castroviejo D, Martin M, Macias M, Escames G, Leon J, Khaldy H, Reiter RJ (2001). Melatonin, mitochondria, and cellular bioenergetics. J. Pineal Res.

[9] Agarwal S, Sohal RS (1995). Differential oxidative damage to mitochondrial proteins during aging. Mech. Ageing Dev.

[10] Andersen HR, Nielsen JB, Nielsen F, Grandjean P (1997). Antioxidative enzyme activities in human erythrocytes. Clin. Chem.

[11] Azari NP, Pettigrew KD, Schapiro MB, Haxby JV, Grady CL, Pietrini P, Salerno JA, Heston LL, Rapoport SI, Horwitz B (1993). Early detection of Alzheimer's disease: a statistical approach using positron emission tomographic data. J Cereb. Blood Flow Metab.

[12] Bachoud-Levi AC, Remy P, Nguyen JP, Brugieres P, Lefaucheur JP, Bourdet C, Baudic S, Gaura V, Maison P, Haddad B, Boisse MF, Grandmougin T, Jeny R, Bartolomeo P, Dalla BG, Degos JD, Lisovoski F, Ergis AM, Pailhous E, Cesaro P, Hantraye P, Peschanski M (2000). Motor and cognitive improvements in patients with Huntington's disease after neural transplantation. Lancet.

[13] Batista CM, Kippin TE, Willaime-Morawek S, Shimabukuro MK, Akamatsu W, van der Kooy D (2006). A progressive and cell non-autonomous increase in striatal neural stem cells in  the Huntington's disease R6/2 mouse. J. Neurosci.

[14] Bender A, Krishnan KJ, Morris CM, Taylor GA, Reeve AK, Perry RH, Jaros E, Hersheson JS, Betts J, Klopstock T, Taylor RW, Turnbull DM (2006). High levels of mitochondrial DNA deletions in substantia nigra neurons in aging and Parkinson disease. Nat. Genet.

[15] Bender A, Schwarzkopf RM, McMillan A, Krishnan KJ, Rieder G, Neumann M, Elstner M, Turnbull DM, Klopstock T (2008). Dopaminergic midbrain neurons are the prime target for mitochondrial DNA deletions. J. Neurol.

[16] Berneburg M, Grether-Beck S, Kurten V, Ruzicka T, Briviba K, Sies H, Krutmann J (1999). Singlet oxygen mediates the UVA-induced generation of the photoaging-associated mitochondrial common deletion. J. Biol. Chem.

[17] Blurton-Jones M, Kitazawa M, Martinez-Coria H, Castello NA, Muller FJ, Loring JF, Yamasaki TR, Poon WW, Green KN, LaFerla FM (2009). Neural stem cells improve cognition *via* BDNF in a transgenic model of Alzheimer disease. Proc. Natl. Acad. Sci. USA.

[18] Bondolfi L, Ermini F, Long JM, Ingram DK, Jucker M (2004). Impact of age and caloric restriction on neurogenesis in the dentate gyrus of C57BL/6 mice. Neurobiol. Aging.

[19] Bonilla E, Tanji K, Hirano M, Vu TH, DiMauro S, Schon EA (1999). Mitochondrial involvement in Alzheimer's disease. Biochim. Biophys. Acta.

[20] Brown GC (2001). Regulation of mitochondrial respiration by nitric oxide inhibition of cytochrome c oxidase. Biochim. Biophys. Acta.

[21] Brusco LI, Marquez M, Cardinali DP (1998). Monozygotic twins with Alzheimer's disease treated with melatonin: Case report. J. Pineal Res.

[22] Brusco LI, Marquez M, Cardinali DP (2000). Melatonin treatment stabilizes chronobiologic and cognitive symptoms in Alzheimer's disease. Neuro Endocrinol. Lett.

[23] Butterfield DA, Howard BJ, Yatin S, Allen KL, Carney JM (1997). Free radical oxidation of brain proteins in accelerated senescence and its modulation by N-tert-butyl-alpha-phenylnitrone. Proc. Natl. Acad. Sci. USA.

[24] Carretero M, Escames G, Lopez LC, Venegas C, Dayoub JC, Garcia L, Acuna-Castroviejo D (2009). Long-term melatonin administration protects brain mitochondria from aging. J. Pineal Res.

[25] Cassina AM, Hodara R, Souza JM, Thomson L, Castro L, Ischiropoulos H, Freeman BA, Radi R (2000). Cytochrome c nitration by peroxynitrite. J. Biol. Chem.

[26] Chen Q, Fischer A, Reagan JD, Yan LJ, Ames BN (1995). Oxidative DNA damage and senescence of human diploid fibroblast cells. Proc. Natl. Acad. Sci. USA.

[27] Chen ST, Chuang JI (1999). The antioxidant melatonin reduces cortical neuronal death after intrastriatal injection of kainate in the rat. Exp. Brain Res.

[28] Cho YM, Kwon S, Pak YK, Seol HW, Choi YM, Park do J, Park KS, Lee HK (2006). Dynamic changes in mitochondrial biogenesis and antioxidant enzymes during the spontaneous differentiation of human embryonic stem cells. Biochem. Biophys. Res. Commun.

[29] Cortopassi GA, Shibata D, Soong NW, Arnheim N (1992). A pattern of accumulation of a somatic deletion of mitochondrial DNA in aging human tissues. Proc. Natl. Acad. Sci. USA.

[30] Curtis MA, Penney EB, Pearson AG, van Roon-Mom WM, Butterworth NJ, Dragunow M, Connor B, Faull RL (2003). Increased cell proliferation and neurogenesis in the adult human Huntington's disease brain. Proc. Natl. Acad. Sci. USA.

[31] Curtis MA, Waldvogel HJ, Synek B, Faull RL (2005). A histochemical and immunohistochemical analysis of the subependymal layer in the normal and Huntington's disease brain. J. Chem. Neuroanat.

[32] Davidzon G, Greene P, Mancuso M, Klos KJ, Ahlskog JE, Hirano M, DiMauro S (2006). Early-onset familial parkinsonism due to POLG mutations. Ann. Neurol.

[33] de Lustig ES, Serra JA, Kohan S, Canziani GA, Famulari AL, Dominguez RO (1993). Copper-zinc superoxide dismutase activity in red blood cells and serum in demented patients and in aging. J. Neurol. Sci.

[34] De BM, Pappas BA (2007). Pinealectomy causes hippocampal CA1 and CA3 cell loss: reversal by melatonin supplementation. Neurobiol. Aging.

[35] DiMauro S, Schon EA (2008). Mitochondrial disorders in the nervous system. Ann. Rev. Neurosci.

[36] DiMauro S, Tanji K, Bonilla E, Pallotti F, Schon EA (2002). Mitochondrial abnormalities in muscle and other aging cells: classification, causes, and effects. Muscle Nerve.

[37] Donovan MH, Yazdani U, Norris RD, Games D, German DC, Eisch AJ (2006). Decreased adult hippocampal neurogenesis in the PDAPP mouse model of Alzheimer's disease. J. Comp. Neurol.

[38] Drapeau E, Nora AD (2008). Stem cell review series: role of neurogenesis in age-related memory disorders. Aging Cell.

[39] Driscoll I, Howard SR, Stone JC, Monfils MH, Tomanek B, Brooks WM, Sutherland RJ (2006). The aging hippocampus: a multi-level analysis in the rat. Neuroscience.

[40] Droge W (2003). Oxidative stress and aging. Adv. Exp. Med. Biol.

[41] Dumont P, Burton M, Chen QM, Gonos ES, Frippiat C, Mazarati JB, Eliaers F, Remacle J, Toussaint O (2000). Induction of replicative senescence biomarkers by sublethal oxidative stresses in normal human fibroblast. Free Radic. Biol. Med.

[42] Escames G, Leon J, Macias M, Khaldy H, Acuna-Castroviejo D (2003). Melatonin counteracts lipopolysaccharide-induced expression and activity of mitochondrial nitric oxide synthase in rats. FASEB J.

[43] Escames G, Lopez LC, Ortiz F, Lopez A, Garcia JA, Ros E, Acuna-Castroviejo D (2007). Attenuation of cardiac mitochondrial dysfunction by melatonin in septic mice. FEBS J.

[44] Escames G, Lopez LC, Ortiz F, Ros E, Acuna-Castroviejo D (2006). Age-dependent lipopolysaccharide-induced iNOS expression and multiorgan failure in rats: effects of melatonin treatment. Exp. Gerontol.

[45] Escames G, Lopez LC, Tapias V, Utrilla P, Reiter RJ, Hitos AB, Leon J, Rodriguez MI, Acuna-Castroviejo D (2006). Melatonin counteracts inducible mitochondrial nitric oxide synthase-dependent mitochondrial dysfunction in skeletal muscle of septic mice. J. Pineal Res.

[46] Escames G, Macias M, Leon J, Garcia J, Khaldy H, Martin M, Vives F, Acuna-Castroviejo D (2001). Calcium-dependent effects of melatonin inhibition of glutamatergic response in rat striatum. J. Neuroendocrinol.

[47] Falcon J, Besseau L, Fuentes M, Sauzet S, Magnanou E, Boeuf G (2009). Structural and functional evolution of the pineal melatonin system in vertebrates. Ann. N .Y. Acad. Sci.

[48] Floyd RA, Hensley K (2002). Oxidative stress in brain aging. Implications for therapeutics of neurodegenerative diseases. Neurobiol. Aging.

[49] Forster MJ, Dubey A, Dawson KM, Stutts WA, Lal H, Sohal RS (1996). Age-related losses of cognitive function and motor skills in mice are associated with oxidative protein damage in the brain. Proc. Natl. Acad. Sci. USA.

[50] Frankel D, Schipper HM (1999). Cysteamine pretreatment of the astroglial substratum (mitochondrial iron sequestration) enhances PC12 cell vulnerability to oxidative injury. Exp. Neurol.

[51] French HM, Reid M, Mamontov P, Simmons RA, Grinspan JB (2009). Oxidative stress disrupts oligodendrocyte maturation. J. Neurosci. Res.

[52] Furio AM, Brusco LI, Cardinali DP (2007). Possible therapeutic value of melatonin in mild cognitive impairment: a retrospective study. J. Pineal Res.

[53] Garbuzova-Davis S, Willing AE, Milliken M, Saporta S, Zigova T, Cahill DW, Sanberg PR (2002). Positive effect of transplantation of hNT neurons (NTera 2/D1 cell-line) in a model of familial amyotrophic lateral sclerosis. Exp. Neurol.

[54] Garcia JJ, Pinol-Ripoll G, Martinez-Ballarin E, Fuentes-Broto L, Miana-Mena FJ, Venegas C, Caballero B, Escames G, Coto-Montes A, Acuna-Castroviejo D (2010). Melatonin reduces membrane rigidity and oxidative damage in the brain of SAMP(8) mice. Neurobiol. Aging.

[55] Gil JM, Mohapel P, Araujo IM, Popovic N, Li JY, Brundin P, Petersen A (2005). Reduced hippocampal neurogenesis in R6/2 transgenic Huntington's disease mice. Neurobiol. Dis.

[56] Goedert M, Spillantini MG (2006). A century of Alzheimer's disease. Science.

[57] Habif S, Mutaf I, Turgan N, Onur E, Duman C, Ozmen D, Bayindir O (2001). Age and gender dependent alterations in the activities of glutathione related enzymes in healthy subjects. Clin. Biochem.

[58] Hagell P, Schrag A, Piccini P, Jahanshahi M, Brown R, Rehncrona S, Widner H, Brundin P, Rothwell JC, Odin P, Wenning GK, Morrish P, Gustavii B, Bjorklund A, Brooks DJ, Marsden CD, Quinn NP, Lindvall O (1999). Sequential bilateral transplantation in Parkinson's disease: effects of the second graft. Brain.

[59] Hardeland R, Poeggeler B (2003). Non-vertebrate melatonin. J. Pineal Res.

[60] Haughey NJ, Liu D, Nath A, Borchard AC, Mattson MP (2002). Disruption of neurogenesis in the subventricular zone of adult mice, and in human cortical neuronal precursor cells in culture, by amyloid beta-peptide: implications for the pathogenesis of Alzheimer's disease. Neuromol. Med.

[61] Haughey NJ, Nath A, Chan SL, Borchard AC, Rao MS, Mattson MP (2002). Disruption of neurogenesis by amyloid beta-peptide, and perturbed neural progenitor cell homeostasis, in models of Alzheimer's disease. J. Neurochem.

[62] Hayakawa M, Hattori K, Sugiyama S, Ozawa T (1992). Age-associated oxygen damage and mutations in mitochondrial DNA in human hearts. Biochem. Biophys. Res. Commun.

[63] Heine VM, Maslam S, Joels M, Lucassen PJ (2004). Prominent decline of newborn cell proliferation, differentiation, and apoptosis in the aging dentate gyrus, in absence of an age-related hypothalamus-pituitary-adrenal axis activation. Neurobiol. Aging.

[64] Hervias I, Beal MF, Manfredi G (2006). Mitochondrial dysfunction and amyotrophic lateral sclerosis. Muscle Nerve.

[65] Hill JM, Switzer RC III (1984). The regional distribution and cellular localization of iron in the rat brain. Neuroscience.

[66] Hoglinger GU, Rizk P, Muriel MP, Duyckaerts C, Oertel WH, Caille I, Hirsch EC (2004). Dopamine depletion impairs precursor cell proliferation in Parkinson disease. Nat. Neurosci.

[67] Horton TM, Graham BH, Corral-Debrinski M, Shoffner JM, Kaufman AE, Beal MF, Wallace DC (1995). Marked increase in mitochondrial DNA deletion levels in the cerebral cortex of Huntington's disease patients. Neurology.

[68] Hosokawa M (2002). A higher oxidative status accelerates senescence and aggravates age-dependent disorders in SAMP strains of mice. Mech. Ageing Dev.

[69] Howell N, Elson JL, Chinnery PF, Turnbull DM (2005). mtDNA mutations and common neurodegenerative disorders. Trends Genet.

[70] Hwang DH, Lee HJ, Park IH, Seok JI, Kim BG, Joo IS, Kim SU (2009). Intrathecal transplantation of human neural stem cells overexpressing VEGF provide behavioral improvement, disease onset delay and survival extension in transgenic ALS mice. Gene Ther.

[71] Iacovitti L, Stull ND, Mishizen A (1999). Neurotransmitters, KCl and antioxidants rescue striatal neurons from apoptotic cell death in culture. Brain Res.

[72] Jin K, LaFevre-Bernt M, Sun Y, Chen S, Gafni J, Crippen D, Logvinova A, Ross CA, Greenberg DA, Ellerby LM (2005). FGF-2 promotes neurogenesis and neuroprotection and prolongs survival in a transgenic mouse model of Huntington's disease. Proc. Natl. Acad. Sci. USA.

[73] Jou MJ, Peng TI, Wu HY, Wei YH (2005). Enhanced generation of mitochondrial reactive oxygen species in cybrids containing 4977-bp mitochondrial DNA deletion. Ann. N Y Acad. Sci.

[74] Jou MJ, Peng TI, Yu PZ, Jou SB, Reiter RJ, Chen JY, Wu HY, Chen CC, Hsu LF (2007). Melatonin protects against common deletion of mitochondrial DNA-augmented mitochondrial oxidative stress and apoptosis. J. Pineal Res.

[75] Kaneko N, Okano H, Sawamoto K (2006). Role of the cholinergic system in regulating survival of newborn neurons in the adult mouse dentate gyrus and olfactory bulb. Genes Cells.

[76] Kaneko N, Sawamoto K (2009). Adult neurogenesis and its alteration under pathological conditions. Neurosci. Res.

[77] Kanemitsu H, Yamauchi H, Komatsu M, Yamamoto S, Okazaki S, Uchida K, Nakayama H (2009). 6-Mercaptopurine (6-MP) induces cell cycle arrest and apoptosis of neural progenitor cells in the developing fetal rat brain. Neurotoxicol. Teratol.

[78] Kanemitsu H, Yamauchi H, Komatsu M, Yamamoto S, Okazaki S, Uchida K, Nakayama H (2009). 6-mercaptopurine (6-MP) induces p53-mediated apoptosis of neural progenitor cells in the developing fetal rodent brain. Neurotoxicol. Teratol.

[79] Karasek M (2004). Melatonin, human aging, and age-related diseases. Exp. Gerontol.

[80] Khaldy H, Escames G, Leon J, Vives F, Luna JD, Acuna-Castroviejo D (2000). Comparative effects of melatonin, L-deprenyl, Trolox and ascorbate in the suppression of hydroxyl radical formation during dopamine autoxidation *in vitro*. J. Pineal Res.

[81] Kilic E, Kilic U, Bacigaluppi M, Guo Z, Abdallah NB, Wolfer DP, Reiter RJ, Hermann DM, Bassetti CL (2008). Delayed melatonin administration promotes neuronal survival, neurogenesis and motor recovery, and attenuates hyperactivity and anxiety after mild focal cerebral ischemia in mice. J. Pineal Res.

[82] Kim SJ, Son TG, Park HR, Park M, Kim MS, Kim HS, Chung HY, Mattson MP, Lee J (2008). Curcumin stimulates proliferation of embryonic neural progenitor cells and neurogenesis in the adult hippocampus. J. Biol. Chem.

[83] Kim SU, de VJ (2009). Stem cell-based cell therapy in neurological diseases: a review. J. Neurosci. Res.

[84] Kirby DM, Rennie KJ, Smulders-Srinivasan TK, Acin-Perez R, Whittington M, Enriquez JA, Trevelyan AJ, Turnbull DM, Lightowlers RN (2009). Transmitochondrial embryonic stem cells containing pathogenic mtDNA mutations are compromised in neuronal differentiation. Cell Prolif.

[85] Kish SJ, Bergeron C, Rajput A, Dozic S, Mastrogiacomo F, Chang LJ, Wilson JM, DiStefano LM, Nobrega JN (1992). Brain cytochrome oxidase in Alzheimer's disease. J. Neurochem.

[86] Kraytsberg Y, Kudryavtseva E, McKee AC, Geula C, Kowall NW, Khrapko K (2006). Mitochondrial DNA deletions are abundant and cause functional impairment in aged human substantia nigra neurons. Nat. Genet.

[87] Kujoth GC, Hiona A, Pugh TD, Someya S, Panzer K, Wohlgemuth SE, Hofer T, Seo AY, Sullivan R, Jobling WA, Morrow JD, Van Remmen H, Sedivy JM, Yamasoba T, Tanokura M, Weindruch R, Leeuwenburgh C, Prolla TA (2005). Mitochondrial DNA mutations, oxidative stress, and apoptosis in mammalian aging. Science.

[88] Lahiri DK, Ge YW, Sharman EH, Bondy SC (2004). Age-related changes in serum melatonin in mice: higher levels of combined melatonin and 6-hydroxymelatonin sulfate in the cerebral cortex than serum, heart, liver and kidney tissues. J. Pineal Res.

[89] Lazic SE, Grote H, Armstrong RJ, Blakemore C, Hannan AJ, van DA, Barker RA (2004). Decreased hippocampal cell proliferation in R6/1 Huntington's mice. Neuroreport.

[90] Leonard JV, Schapira AH (2000). Mitochondrial respiratory chain disorders II: neurodegenerative disorders and nuclear gene defects. Lancet.

[91] Lichtenwalner RJ, Forbes ME, Bennett SA, Lynch CD, Sonntag WE, Riddle DR (2001). Intracerebroventricular infusion of insulin-like growth factor-I ameliorates the age-related decline in hippocampal neurogenesis. Neuroscience.

[92] Liu J, Mori A (1993). Age-associated changes in superoxide dismutase activity, thiobarbituric acid reactivity and reduced glutathione level in the brain and liver in senescence accelerated mice (SAM): a comparison with ddY mice. Mech. Ageing Dev.

[93] Loeb LA, Wallace DC, Martin GM (2005). The mitochondrial theory of aging and its relationship to reactive oxygen species damage and somatic mtDNA mutations. Proc. Natl. Acad. Sci. USA.

[94] Lonergan T, Bavister B, Brenner C (2007). Mitochondria in stem cells. Mitochondrion.

[95] Lopez A, Garcia JA, Escames G, Venegas C, Ortiz F, Lopez LC, Acuna-Castroviejo D (2009). Melatonin protects the mitochondria from oxidative damage reducing oxygen consumption, membrane potential, and superoxide anion production. J. Pineal Res.

[96] Lopez LC, Escames G, Ortiz F, Ros E, Acuna-Castroviejo D (2006). Melatonin restores the mitochondrial production of ATP in septic mice. Neuro Endocrinol. Lett.

[97] Lopez LC, Escames G, Tapias V, Utrilla P, Leon J, Acuna-Castroviejo D (2006). Identification of an inducible nitric oxide synthase in diaphragm mitochondria from septic mice: its relation with mitochondrial dysfunction and prevention by melatonin. Int. J. Biochem. Cell Biol.

[98] Luoma P, Melberg A, Rinne JO, Kaukonen JA, Nupponen NN, Chalmers RM, Oldfors A, Rautakorpi I, Peltonen L, Majamaa K, Somer H, Suomalainen A (2004). Parkinsonism, premature menopause, and mitochondrial DNA polymerase gamma mutations: clinical and molecular genetic study. Lancet.

[99] Ma YS, Wu SB, Lee WY, Cheng JS, Wei YH (2009). Response to the increase of oxidative stress and mutation of mitochondrial DNA in aging. Biochim. Biophys. Acta.

[100] Madhavan L, Ourednik V, Ourednik J (2008). Neural stem/progenitor cells initiate the formation of cellular networks that provide neuroprotection by growth factor-modulated antioxidant expression. Stem Cells.

[101] Manda K, Ueno M, Anzai K (2009). Cranial irradiation-induced inhibition of neurogenesis in hippocampal dentate gyrus of adult mice: attenuation by melatonin pretreatment. J. Pineal Res.

[102] Marzani B, Felzani G, Bellomo RG, Vecchiet J, Marzatico F (2005). Human muscle aging: ROS-mediated alterations in rectus abdominis and vastus lateralis muscles. Exp. Gerontol.

[103] Mason RP, Leeds PR, Jacob RF, Hough CJ, Zhang KG, Mason PE, Chuang DM (1999). Inhibition of excessive neuronal apoptosis by the calcium antagonist amlodipine and antioxidants in cerebellar granule cells. J. Neurochem.

[104] Mastrogiacomo F, Bergeron C, Kish SJ (1993). Brain alpha-ketoglutarate dehydrogenase complex activity in Alzheimer's disease. J. Neurochem.

[105] Matsugo S, Kitagawa T, Minami S, Esashi Y, Oomura Y, Tokumaru S, Kojo S, Matsushima K, Sasaki K (2000). Age-dependent changes in lipid peroxide levels in peripheral organs, but not in brain, in senescence-accelerated mice. Neurosci. Lett.

[106] Mayo JC, Sainz RM, Antolin I, Rodriguez C (1999). Ultrastructural confirmation of neuronal protection by melatonin against the neurotoxin 6-hydroxydopamine cell damage. Brain Res.

[107] Mazzini L, Ferrero I, Luparello V, Rustichelli D, Gunetti M, Mareschi K, Testa L, Stecco A, Tarletti R, Miglioretti M, Fava E, Nasuelli N, Cisari C, Massara M, Vercelli R, Oggioni GD, Carriero A, Cantello R, Monaco F, Fagioli F (2009). Mesenchymal stem cell transplantation in amyotrophic lateral sclerosis: A Phase I clinical trial. Exp. Neurol.

[108] Michikawa Y, Mazzucchelli F, Bresolin N, Scarlato G, Attardi G (1999). Aging-dependent large accumulation of point mutations in the human mtDNA control region for replication. Science.

[109] Miquel J (1998). An update on the oxygen stress-mitochondrial mutation theory of aging: genetic and evolutionary implications. Exp. Gerontol.

[110] Miranda S, Opazo C, Larrondo LF, Munoz FJ, Ruiz F, Leighton F, Inestrosa NC (2000). The role of oxidative stress in the toxicity induced by amyloid beta-peptide in Alzheimer's disease. Prog. Neurobiol.

[111] Miyako K, Kai Y, Irie T, Takeshige K, Kang D (1997). The content of intracellular mitochondrial DNA is decreased by 1-methyl-4-phenylpyridinium ion (MPP+). J. Biol. Chem.

[112] Moriya T, Horie N, Mitome M, Shinohara K (2007). Melatonin influences the proliferative and differentiative activity of neural stem cells. J. Pineal Res.

[113] Nakao N, Itakura T (2000). Fetal tissue transplants in animal models of Huntington's disease: the effects on damaged neuronal circuitry and behavioral deficits. Prog. Neurobiol.

[114] Navarro A, Sanchez Del Pino MJ, Gomez C, Peralta JL, Boveris A (2002). Behavioral dysfunction, brain oxidative stress, and impaired mitochondrial electron transfer in aging mice. Am J Physiol Regul. Integr. Comp Physiol.

[115] Nichols NR, Zieba M, Bye N (2001). Do glucocorticoids contribute to brain aging?. Brain Res. Brain Res. Rev.

[116] Nicolle MM, Gonzalez J, Sugaya K, Baskerville KA, Bryan D, Lund K, Gallagher M, McKinney M (2001). Signatures of hippocampal oxidative stress in aged spatial learning-impaired rodents. Neuroscience.

[117] Nomura Y, Wang BX, Qi SB, Namba T, Kaneko S (1989). Biochemical changes related to aging in the senescence-accelerated mouse. Exp. Gerontol.

[118] Oh SK, Kim HS, Ahn HJ, Seol HW, Kim YY, Park YB, Yoon CJ, Kim DW, Kim SH, Moon SY (2005). Derivation and characterization of new human embryonic stem cell lines: SNUhES1, SNUhES2, and SNUhES3. Stem Cells.

[119] Olcese JM, Cao C, Mori T, Mamcarz MB, Maxwell A, Runfeldt MJ, Wang L, Zhang C, Lin X, Zhang G, Arendash GW (2009). Protection against cognitive deficits and markers of neurodegeneration by long-term oral administration of melatonin in a transgenic model of Alzheimer disease. J. Pineal Res.

[120] Ortiz GG, Crespo-Lopez ME, Moran-Moguel C, Garcia JJ, Reiter RJ, Acuna-Castroviejo D (2001). Protective role of melatonin against MPTP-induced mouse brain cell DNA fragmentation and apoptosis in vivo. Neuro Endocrinol. Lett.

[121] Pansarasa O, Bertorelli L, Vecchiet J, Felzani G, Marzatico F (1999). Age-dependent changes of antioxidant activities and markers of free radical damage in human skeletal muscle. Free Radic. Biol. Med.

[122] Pappolla MA, Chyan YJ, Poeggeler B, Bozner P, Ghiso J, LeDoux SP, Wilson GL (1999). Alzheimer beta protein mediated oxidative damage of mitochondrial DNA: prevention by melatonin. J. Pineal Res.

[123] Pappolla MA, Chyan YJ, Poeggeler B, Frangione B, Wilson G, Ghiso J, Reiter RJ (2000). An assessment of the antioxidant and the antiamyloidogenic properties of melatonin: implications for Alzheimer's disease. J. Neural. Transm.

[124] Pappolla MA, Sos M, Omar RA, Bick RJ, Hickson-Bick DL, Reiter RJ, Efthimiopoulos S, Robakis NK (1997). Melatonin prevents death of neuroblastoma cells exposed to the Alzheimer amyloid peptide. J. Neurosci.

[125] Petrosillo G, Fattoretti P, Matera M, Ruggiero FM, Bertoni-Freddari C, Paradies G (2008). Melatonin prevents age-related mitochondrial dysfunction in rat brain *via* cardiolipin protection. Rejuvenation. Res.

[126] Poderoso JJ, Carreras MC, Lisdero C, Riobo N, Schopfer F, Boveris A (1996). Nitric oxide inhibits electron transfer and increases superoxide radical production in rat heart mitochondria and submitochondrial particles. Arch. Biochem. Biophys.

[127] Ramirez-Rodriguez G, Klempin F, Babu H, Benitez-King G, Kempermann G (2009). Melatonin Modulates Cell Survival of New Neurons in the Hippocampus of Adult Mice. Neuropsychopharmacology.

[128] Rao MS, Hattiangady B, Abdel-Rahman A, Stanley DP, Shetty AK (2005). Newly born cells in the ageing dentate gyrus display normal migration, survival and neuronal fate choice but endure retarded early maturation. Eur. J. Neurosci.

[129] Rao MS, Hattiangady B, Shetty AK (2006). The window and mechanisms of major age-related decline in the production of new neurons within the dentate gyrus of the hippocampus. Aging Cell.

[130] Reddy PH, Mao P, Manczak M (2009). Mitochondrial structural and functional dynamics in Huntington's disease. Brain Res. Rev.

[131] Redmond DE, Bjugstad KB, Teng YD, Ourednik V, Ourednik J, Wakeman DR, Parsons XH, Gonzalez R, Blanchard BC, Kim SU, Gu Z, Lipton SA, Markakis EA, Roth RH, Elsworth JD, Sladek JR, Sidman RL, Snyder EY (2007). Behavioral improvement in a primate Parkinson's model is associated with multiple homeostatic effects of human neural stem cells. Proc. Natl. Acad. Sci. USA.

[132] Reiter RJ (1991). Melatonin: the chemical expression of darkness. Mol. Cell Endocrinol.

[133] Reiter RJ (1994). Pineal function during aging: attenuation of the melatonin rhythm and its neurobiological consequences. Acta Neurobiol. Exp (Wars.).

[134] Reiter RJ (1995). Oxidative processes and antioxidative defense mechanisms in the aging brain. FASEB J.

[135] Reiter RJ, Acuna-Castroviejo D, Tan DX, Burkhardt S (2001). Free radical-mediated molecular damage. Mechanisms for the protective actions of melatonin in the central nervous system. Ann. N Y Acad. Sci.

[136] Reiter RJ, Tan DX, Mayo JC, Sainz RM, Leon J, Czarnocki Z (2003). Melatonin as an antioxidant: biochemical mechanisms and pathophysiological implications in humans. Acta Biochim. Pol.

[137] Rennie K, De BM, Pappas BA (2009). Melatonin promotes neurogenesis in dentate gyrus in the pinealectomized rat. J. Pineal Res.

[138] Rodriguez MI, Carretero M, Escames G, Lopez LC, Maldonado MD, Tan DX, Reiter RJ, Acuna-Castroviejo D (2007). Chronic melatonin treatment prevents age-dependent cardiac mitochondrial dysfunction in senescence-accelerated mice. Free Radic. Res.

[139] Rodriguez MI, Escames G, Lopez LC, Garcia JA, Ortiz F, Lopez A, Acuna-Castroviejo D (2007). Melatonin administration prevents cardiac and diaphragmatic mitochondrial oxidative damage in senescence-accelerated mice. J. Endocrinol.

[140] Rodriguez MI, Escames G, Lopez LC, Lopez A, Garcia JA, Ortiz F, Acuna-Castroviejo D (2007). Chronic melatonin treatment reduces the age-dependent inflammatory process in senescence-accelerated mice. J. Pineal Res.

[141] Sauer H, Wartenberg M (2005). Reactive oxygen species as signaling molecules in cardiovascular differentiation of embryonic stem cells and tumor-induced angiogenesis. Antioxid. Redox Signal.

[142] Schapira AH (1999). Mitochondrial involvement in Parkinson's disease, Huntington's disease, hereditary spastic paraplegia and Friedreich's ataxia. Biochim. Biophys. Acta.

[143] Sharma RK, Zhou Q, Netland PA (2008). Effect of oxidative preconditioning on neural progenitor cells. Brain Res.

[144] Sheu KF, Kim YT, Blass JP, Weksler ME (1985). An immunochemical study of the pyruvate dehydrogenase deficit in Alzheimer's disease brain. Ann. Neurol.

[145] St John JC, Ramalho-Santos J, Gray HL, Petrosko P, Rawe VY, Navara CS, Simerly CR, Schatten GP (2005). The expression of mitochondrial DNA transcription factors during early cardiomyocyte *in vitro* differentiation from human embryonic stem cells. Cloning Stem Cells.

[146] Stefulj J, Hortner M, Ghosh M, Schauenstein K, Rinner I, Wolfler A, Semmler J, Liebmann PM (2001). Gene expression of the key enzymes of melatonin synthesis in extrapineal tissues of the rat. J. Pineal Res.

[147] Takagi Y, Takahashi J, Saiki H, Morizane A, Hayashi T, Kishi Y, Fukuda H, Okamoto Y, Koyanagi M, Ideguchi M, Hayashi H, Imazato T, Kawasaki H, Suemori H, Omachi S, Iida H, Itoh N, Nakatsuji N, Sasai Y, Hashimoto N (2005). Dopaminergic neurons generated from monkey embryonic stem cells function in a Parkinson primate model. J. Clin. Invest.

[148] Takeda T (1999). Senescence-accelerated mouse (SAM): a biogerontological resource in aging research. Neurobiol. Aging.

[149] Takeda T, Hosokawa M, Higuchi K (1997). Senescence-accelerated mouse (SAM): a novel murine model of senescence. Exp. Gerontol.

[150] Takeda T, Hosokawa M, Takeshita S, Irino M, Higuchi K, Matsushita T, Tomita Y, Yasuhira K, Hamamoto H, Shimizu K, Ishii M, Yamamuro T (1981). A new murine model of accelerated senescence. Mech. Ageing Dev.

[151] Tamm C, Sabri F, Ceccatelli S (2008). Mitochondrial-mediated apoptosis in neural stem cells exposed to manganese. Toxicol. Sci.

[152] Tan DX, Manchester LC, Hardeland R, Lopez-Burillo S, Mayo JC, Sainz RM, Reiter RJ (2003). Melatonin: a hormone, a tissue factor, an autocoid, a paracoid, and an antioxidant vitamin. J. Pineal Res.

[153] Tapias V, Escames G, Lopez LC, Lopez A, Camacho E, Carrion MD, Entrena A, Gallo MA, Espinosa A, Acuna-Castroviejo D (2009). Melatonin and its brain metabolite N(1)-acetyl-5-methoxykynuramine prevent mitochondrial nitric oxide synthase induction in parkinsonian mice. J. Neurosci. Res.

[154] Tian C, Murrin LC, Zheng JC (2009). Mitochondrial fragmentation is involved in methamphetamine-induced cell death in rat hippocampal neural progenitor cells. PLoS One.

[155] Torreilles F, Salman-Tabcheh S, Guerin M, Torreilles J (1999). Neurodegenerative disorders: the role of peroxynitrite. Brain Res. Brain Res. Rev.

[156] Trifunovic A, Wredenberg A, Falkenberg M, Spelbrink JN, Rovio AT, Bruder CE, Bohlooly YM, Gidlof S, Oldfors A, Wibom R, Tornell J, Jacobs HT, Larsson NG (2004). Premature ageing in mice expressing defective mitochondrial DNA polymerase. Nature.

[157] Tunez I, Montilla P, Del Carmen MM, Feijoo M, Salcedo M (2004). Protective effect of melatonin on 3-nitropropionic acid-induced oxidative stress in synaptosomes in an animal model of Huntington's disease. J. Pineal Res.

[158] van DA, Blakemore C, Deacon R, York D, Hannan AJ (2000). Delaying the onset of Huntington's in mice. Nature.

[159] Vermulst M, Wanagat J, Kujoth GC, Bielas JH, Rabinovitch PS, Prolla TA, Loeb LA (2008). DNA deletions and clonal mutations drive premature aging in mitochondrial mutator mice. Nat. Genet.

[160] Wang Y, Michikawa Y, Mallidis C, Bai Y, Woodhouse L, Yarasheski KE, Miller CA, Askanas V, Engel WK, Bhasin S, Attardi G (2001). Muscle-specific mutations accumulate with aging in critical human mtDNA control sites for replication. Proc. Natl. Acad. Sci. USA.

[161] Wei YH (1998). Oxidative stress and mitochondrial DNA mutations in human aging. Proc. Soc. Exp. Biol. Med.

[162] Wei YH, Lu CY, Lee HC, Pang CY, Ma YS (1998). Oxidative damage and mutation to mitochondrial DNA and age-dependent decline of mitochondrial respiratory function. Ann. N. Y. Acad. Sci.

[163] Weishaupt JH, Bartels C, Polking E, Dietrich J, Rohde G, Poeggeler B, Mertens N, Sperling S, Bohn M, Huther G, Schneider A, Bach A, Siren AL, Hardeland R, Bahr M, Nave KA, Ehrenreich H (2006). Reduced oxidative damage in ALS by high-dose enteral melatonin treatment. J. Pineal Res.

[164] Wen PH, Hof PR, Chen X, Gluck K, Austin G, Younkin SG, Younkin LH, DeGasperi R, Gama Sosa MA, Robakis NK, Haroutunian V, Elder GA (2004). The presenilin-1 familial Alzheimer disease mutant P117L impairs neurogenesis in the hippocampus of adult mice. Exp. Neurol.

[165] Wu YH, Feenstra MG, Zhou JN, Liu RY, Torano JS, Van Kan HJ, Fischer DF, Ravid R, Swaab DF (2003). Molecular changes underlying reduced pineal melatonin levels in Alzheimer disease: alterations in preclinical and clinical stages. J. Clin. Endocrinol. Metab.

[166] Xu L, Yan J, Chen D, Welsh AM, Hazel T, Johe K, Hatfield G, Koliatsos VE (2006). Human neural stem cell grafts ameliorate motor neuron disease in SOD-1 transgenic rats. Transplantation.

[167] Yagita Y, Kitagawa K, Ohtsuki T, Takasawa K, Miyata T, Okano H, Hori M, Matsumoto M (2001). Neurogenesis by progenitor cells in the ischemic adult rat hippocampus. Stroke.

[168] Yan LJ, Levine RL, Sohal RS (1997). Oxidative damage during aging targets mitochondrial aconitase. Proc. Natl. Acad. Sci. USA.

[169] Yan LJ, Sohal RS (1998). Mitochondrial adenine nucleotide translocase is modified oxidatively during aging. Proc. Natl. Acad. Sci. USA.

[170] Yang CR, Yu RK (2009). Intracerebral transplantation of neural stem cells combined with trehalose ingestion alleviates pathology in a mouse model of Huntington's disease. J. Neurosci. Res.

[171] Yarian CS, Rebrin I, Sohal RS (2005). Aconitase and ATP synthase are targets of malondialdehyde modification and undergo an age-related decrease in activity in mouse heart mitochondria. Biochem. Biophys. Res. Commun.

[172] Yasuhara T, Matsukawa N, Hara K, Yu G, Xu L, Maki M, Kim SU, Borlongan CV (2006). Transplantation of human neural stem cells exerts neuroprotection in a rat model of Parkinson's disease. J. Neurosci.

[173] Zaman V, Shetty AK (2002). Combined neurotrophic supplementation and caspase inhibition enhances survival of fetal hippocampal CA3 cell grafts in lesioned CA3 region of the aging hippocampus. Neuroscience.

[174] Zhou JN, Liu RY, Kamphorst W, Hofman MA, Swaab DF (2003). Early neuropathological Alzheimer's changes in aged individuals are accompanied by decreased cerebrospinal fluid melatonin levels. J. Pineal Res.

[175] Zitnik G, Martin GM (2002). Age-related decline in neurogenesis: old cells or old environment?. J. Neurosci. Res.

